# Re: letter to the editor for the article “Health-related quality of life following salvage radical prostatectomy for recurrent prostate cancer after radiotherapy or focal therapy”

**DOI:** 10.1007/s00345-024-05255-z

**Published:** 2024-09-27

**Authors:** Severin Rodler, Christian Stief, Thilo Westhofen

**Affiliations:** 1 Department of Urology, LMU University Hospital, Munich, Germany; 2 Department of Urology, University Hospital of Schleswig-Holstein, Arnold-Heller-Straße 3, 24105 Kiel, Germany

**Keywords:** Quality of life, Salvage radical prostatectomy, Long-term outcome

Dear editor,

We thank *Lu et al.* [1] for their letter in response to our study on health related quality of life in patients undergoing salvage prostatectomy [2]. The letter highlights some important aspects of our work and the work of others in this exciting field. Salvage prostatectomy remains a partially controversial and individualised treatment option for patients with radiorecurrent prostate cancer [3].

Alternative treatment options to salvage prostatectomy include a wide range of therapeutic approaches ranging from high-intensity focused ultrasound (HIFU), cryotherapy, low–dose-rate (LDR) and high-dose-rate (HDR) brachytherapy as well as stereotactic body radiotherapy (SBRT) [4]. However, as missed by Lu et al., the most important comparator is androgen deprivation therapy that is recommended by guidelines and is used in clinical practice for the majority of patients in this treatment setting [5]. As outlined in our study, a direct comparison is difficult, as randomized controlled trials are difficult to design in this setting. Therefore, real-world evidence with clearly described patient characteristics is of high importance to counsel patients with similar background accordingly and to direct them for the most appropriate treatment. We focused on HRQoL as this endpoint is of increasing importance in this treatment setting and perspective on HRQOL might be different for patients with prior focal therapy and radiotherapy that had opted for different treatment options based on potential adverse events and HRQoL.

The authors refer to various factors that impact HRQoL. Interestingly, the authors emphasize digital health literacy as important. We agree that implementation of technology into healthcare and understanding uptake of technology especially for prostate cancer treatment is of high importance [6]. We further agree that there is a variety of factors that are associated with differences in HRQoL. However, for the current study we aimed to describe HRQoL, functionional and oncological outcomes of salvage prostatectomy. In addition, we focused on the primary treatment, time between primary treatment and salvage prostatectomy, erectile function at baseline, continence recovery, biochemical recurrence, and comorbidities as important variables. Adding additional factors in the future and understanding factors that are associated with improved HRQoL as mentioned in the comment is important. However, understanding not only correlations but also causative factors will help to address changes in HRQoL and to provide personalized treatment to patients.

It is important to note that both cohorts in our study differ because of two distinct options in initial choice of treatment and therefore a different adverse event spectrum. This is certainly the most important factor limiting direct comparisons that is not addressed in this comment that is also reflected in age differences at baseline in our study cohort.

In conclusion, we agree that HRQoL gains interest as an important outcome of salvage radical prostatectomy but has to be balanced with functional and oncological outcomes as described in our study. In the long term, oncological results will undoubtedly impact future application of HRQoL with recurrence-free survival as one main driver of high HRQoL. Importantly, further factors leading to higher HRQoL might be identified in the future.

## Data Availability

No datasets were generated or analysed during the current study.
